# Editorial: DNA repair and interventions in aging

**DOI:** 10.3389/fragi.2023.1306463

**Published:** 2023-12-05

**Authors:** Robert M. Brosh, Alexey Moskalev, Vera Gorbunova

**Affiliations:** ^1^ Helicases and Genomic Integrity Section, Translational Gerontology Branch, National Institute on Aging, NIH, Baltimore, MD, United States; ^2^ Institute of Biology, Komi Science Center (RAS), Russian Academy of Sciences, Syktyvkar, Russia; ^3^ Departments of Biology and Medicine, University of Rochester, Rochester, NY, United States

**Keywords:** DNA repair, aging, epigenetic, oxidative stress, cockayne syndrome, NAD^+^, mouse model, biomarker

## *Frontiers in Aging* Research Topic on DNA repair and interventions in aging

*Frontiers in Aging* has recently published a Research Topic on the Research Topic DNA Repair and Interventions in Aging DNA Repair and Interventions in Aging | Frontiers Research Topic (frontiersin.org). The purpose of the Research Topic is to highlight genomic instability as a primary hallmark of aging. The premise that genes implicated in the DNA damage response and DNA repair play a vital role in determining lifespan is reinforced by findings that a number of hereditary accelerated aging disorders are linked to mutations in genes that are responsible for maintenance of chromosomal integrity. In addition, studies of model genetic organisms including mice which serve as valuable preclinical models for human aging support the hypothesis that DNA repair modulation may become a viable means to affect aging and pose a source of intervention for age-related diseases. On the other hand, modulation of expression or function for DNA repair genetically or pharmacologically may yield new insights to not only aging processes but also carcinogenesis. We hope the readers of this *Frontiers in Aging* SI will enjoy reading this unique compilation of both research and review articles on these hot topics. Below, we have provided brief and teasing summaries of the featured articles in the Research Topic.

## Adaptation of DNA methylation aging clock from blood to saliva with cell type variability adjustment

There is tremendous interest in biomarkers of aging from both a basic science and clinical perspective. Aging researchers and clinicians, as well as the public at large, are captivated by new advances that provide evidence for a model in which characteristics and phenotypes of aging can be quantified in a manner that can be used to predict the biological age of an individual in comparison to chronological age, i.e., time elapsed since birth. For an informed and very current perspective, see Moqri et al. (2023). Determining reliable parameters in aging biology opens the door for carefully monitoring therapeutic longevity interventions, building upon the working model that “geroprotectors” exist that can help to suppress biomarkers of aging, thereby increasing healthspan and lifespan of individuals in the population. A formidable challenge is to identify useful and reliable biomarkers to measure aging clocks that accurately tracks the pace of aging in genetic models and even human beings. One particular molecular biomarker of aging is epigenomics that can serve as a useful predictive, prognostic or response parameter which satisfies basic criteria for ideality in its application (Milicic et al., 2023). A good example in this class is DNA methylation that regulates tissue-specific gene expression and can be readily assessed by molecular analysis of biologically and clinically accessible samples including blood and saliva. However, the utility of DNA methylation may be greatly influenced by its tissue source, requiring sophisticated advances to translate the genomic epigenetic pattern in a quantitative manner to predict the aging clock, biological versus chronological pace of aging.

In their report featured in the *Frontiers in Aging* Research Topic (SI) DNA Repair and Interventions in Aging, Galkin et al. describe the application of a reference-based tool to enable a rigorous epigenetic analysis of the DNA methylation (DNAm) aging clock using saliva samples as opposed to blood (Galkin et al.). The DeepMAge aging clock was previously trained on a Research Topic of blood DNAm profiles (Galkin et al., 2021b). In this new work, cell type variability adjustments were applied to saliva samples to improve age prediction accuracy using DeepMAge. By employing a cell type deconvolution to determine cell type composition and ElasticNet fitter to achieve optimal regression parameters, the researchers were able to significantly improve the ability of DeepMAge to predict chronological age in saliva samples from over 20 years to less than 5 years without retraining the data set in the deep learning model. More generally, the cell-type deconvolution approach employed may serve as a model to expand the practical application of the aging clocks, as exemplified by the transition from blood to saliva analysis.

## Genetic risk and systemic oxidative stress

While endogenous or exogenously induced reactive oxygen species (ROS) are known to damage macromolecules (nucleic acids, proteins, and lipids) and alter cellular dynamics and homeostasis contributing to tissue and organs decline in model genetic organisms and humans, the mechanisms that respond to, suppress, or repair oxidatively damaged molecules is complex in their different pathways and regulation are an active area of study. Genetic predisposition, environmental exposure and lifestyle choices all play a significant role in oxidative stress, evidenced by specific biomarkers in human populations. Of the numerous genes implicated in oxidative stress, selected genes encoding anti-oxidant enzymes (AE) and DNA base excision repair (BER) proteins were investigated by Mao et al. in a pilot study for single nucleotide polymorphisms (SNPs) and their contribution to an oxidative stress biomarker, namely plasma F2-isoprostanes (FiP) concentrations (Mao et al.). In addition, a biomarker-weighted balance score (OBS) involving anti- and pro-oxidant dietary and lifestyle exposures was calculated. The human subjects of the two pooled cross-sectional studies were in the age range of 30–74 years, white race, and free of cancer or inflammatory bowel disease. Their results suggested that the genotypes of certain DNA BER genes and an AE gene are associated with plasma FiP concentrations; however, examination of larger populations and additional oxidative stress genes would further strengthen the findings. Further efforts to correlate genetic risk scores in normal populations with systemic oxidative stress biomarkers are warranted as we come to appreciate the complex inter-relationships of heredity, environmental exposure, and lifestyle choices in health, aging, disease, and cancer.

## Tipping the balance: cockayne syndrome genes implicated in premature aging and cancer

DNA repair and transcriptional regulation are complex cellular processes, mediated by a myriad of proteins, that are critical for suppression of age-related diseases, neurodegeneration, and cancer. Therefore, it is not unexpected that hereditary mutations in specific genes encoding factors essential to the DNA damage response and gene expression are linked to diseases of premature aging but also associated with a spectrum of cancers. Cockayne Syndrome (CS) caused by bi-allelic mutations in the *CSA* or *CSB* genes (and additional genetic complementation groups) has a hallmark defect on transcription-coupled repair (TCR) of DNA damage. Paccosi et al. have contributed to the *Frontiers in Aging* SI an article on CSA and CSB that provides a comprehensive and insightful perspective of how CSA and CSB loss of function is implicated in the molecular pathology of CS, and conversely how up-regulation of CSA and CSB plays a role in carcinogenesis (Paccosi et al.). This review provides not only an updated summary of the molecular functions of CSA/CSB in TCR and transcriptional regulation that contribute to apoptosis and senescence, but also how elevated expression of the normal (unmutated) *CSA* and *CSB* genes confers a pro-survival cellular growth advantage that favors cancer development and progression. A special section of the review features proposed research directions to develop therapeutic strategies for CS patients and potential avenues to target CSA and CSB for cancer treatment. Readers should find this contribution useful for appreciating the advances that have been made to characterize the complex DNA repair/transcription disorder caused by mutations in *CSA* and *CSB*, and how their fine-tuned regulation is important in cancer.

## Value of short-lived DNA repair mutant mouse models for drug intervention screens

With the rapidly increasing public interest in pharmaceutical drugs proposed to have anti-aging properties, the challenge to screen and characterize such compounds in a methodical scientific-based regime in translational models is of highest importance. Mouse models remain a gold standard for preclinical studies of drugs that eventually are selected for or rejected from further clinical trials. Of high priority and utility has been certain DNA repair deficient mutant mouse models that display features of accelerated aging in many (but not all) tissues and organs. Progeroid mouse models with inactivating mutations in the *ERCC1* or *XPG* nucleotide excision repair genes are two such models that have been intensely studied and appreciated. In the Research Topic of *Frontiers in Aging* dedicated to DNA repair and aging interventions, Birkisdottir and collaborators have tested a panel of compounds that impact nutrient sensing, inflammation, mitochondrial processes, glucose homeostasis, and nicontinamide adenine dinucleotide (NAD^+^) metabolism for their effects on short lifespan and early onset of neurological degeneration in the mice mutated for *ERCC1* or *XPG* (Birkisdóttir et al.). Of these drugs, notably the two NAD^+^ precursors nicotinamide riboside (NR) and nicotinic acid (NA) were beneficial, in accord with other studies indicative of the positive influence of NAD^+^ on DNA repair and associated phenotypes.

## Perspective on recently published cell paper “loss of epigenetic information as a cause of aging”

While it is widely accepted that DNA damage is a causative force of aging, the mechanistic pathway (s) whereby the accompanying declined function of tissues and cells in mammals remains a topic of debate. It has been proposed that double-strand breaks (DSBs) are a driver of aging phenotypes, but how the signals of this highly recombinogenic and toxic lesion are transmitted to loss of cellular structure and function has been puzzling, at least in part due to the lack of a good experimental system. As a part of the *Frontiers in Aging* SI on DNA Repair and Interventions in Aging, Schaffer et al. provide their perspective on a recent Cell paper from the Sinclair lab and collaborators who developed a clever DSB-inducible system at the cellular and organismal (mouse) level to reveal that epigenetic changes to genomic DNA play a key role in aging (Yang et al., 2023). Schaffer et al. discuss the key advances and limitations of the Yang et al. *Cell* paper, as well as the perceived implications for healthspan and how epigenetic reversal of aging might be a means for re-differentiation of cells to protect the affected tissues and organs of a chronologically aging organism to combat disease-related processes.

## Summary

Like many of its *Frontiers in Aging* special Research Topic, the SI focused on DNA Repair and Interventions provides readers the opportunity to dive into topics and latest developments that have implications for understanding the biology of aging but also clinical implications for biomarkers and suppression of malevolent processes that affect molecular processes, cellular phenotypes, and decline at the tissue and organ levels. The Guest Editors for this *Frontiers in Aging* SI thank the authors for their contributions which we hope will advance the aging research community through communication of new ideas and critical assessments.
